# *N*,*N*-Diethyl-4-[(*E*)-(pyridin-3-yl)diazen­yl]aniline

**DOI:** 10.1107/S1600536813019545

**Published:** 2013-07-20

**Authors:** Sergiu Draguta, Evgeniya Leonova, Maria Fokina, Igor Denisyuk, Tatiana V. Timofeeva

**Affiliations:** a D. Ghitu Institute of Electronic Engineering and Nanotechnologies, 3/3 Academy Street, MD-2028, Chisinau, Republic of Moldova; b Department of Chemistry and Biology, New Mexico Highlands University, 803 University Avenue, Las Vegas, NM 87701, USA; c Saint-Petersburg National Research University of Information Technologies, Mechanics and Optics, Kronverkskiy Prospekt 49, 197101 Saint Petersburg, Russian Federation

## Abstract

The mol­ecule of the title compound, C_15_H_18_N_4_, adopts a *trans* conformation with respect to the diazo N=N bond. The dihedral angle between the benzene and pyridine rings in the mol­ecule is 8.03 (5)°. In the crystal, a weak C—H⋯π inter­action arranges the mol­ecules into a corrugated ribbon, with an anti­parallel orientation of neighboring mol­ecules propagating in the [100] direction.

## Related literature

For details of the synthesis, see: Peor *et al.* (2008 ▶). For nonlinear optical properties of stilbene derivatives, see: Forrest *et al.* (1996 ▶). For the comparision of nonlinear optical properties of stilbene and diazo derivatives, see: Chemla & Zyss (1987 ▶); Morley (1995 ▶). For second-harmonic generation in the *P*2_1_2_1_2_1_ space group, see: Rivera *et al.* (2006 ▶). For the distribution of endocyclic angles in pyridine derivatives, see: Draguta *et al.* (2012 ▶).
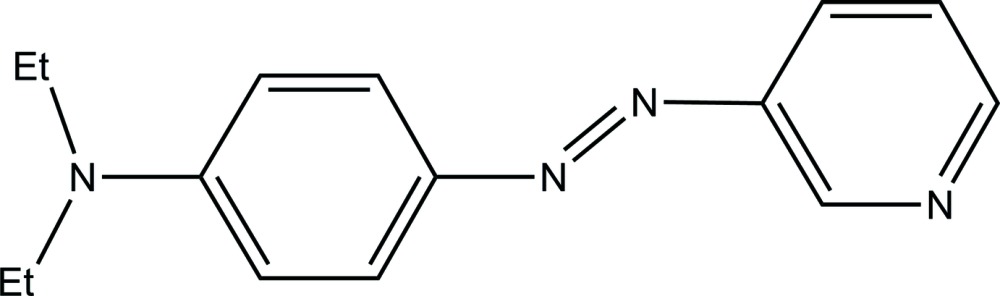


## Experimental

### 

#### Crystal data

C_15_H_18_N_4_
*M* *_r_* = 254.33
Orthorhombic, 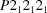

*a* = 7.4332 (7) Å
*b* = 9.1093 (8) Å
*c* = 20.1946 (19) Å
*V* = 1367.4 (2) Å^3^
*Z* = 4
Mo *K*α radiation
μ = 0.08 mm^−1^
*T* = 100 K
0.30 × 0.25 × 0.20 mm

#### Data collection

Bruker APEXII CCD diffractometer
Absorption correction: multi-scan (*SADABS*; Sheldrick, 2003 ▶) *T*_min_ = 0.977, *T*_max_ = 0.985
16318 measured reflections
4195 independent reflections
4012 reflections with *I* > 2σ(*I*)
*R*_int_ = 0.022

#### Refinement

*R*[*F* ^2^ > 2σ(*F* ^2^)] = 0.046
*wR*(*F* ^2^) = 0.113
*S* = 1.00
4195 reflections
174 parameters
H-atom parameters constrained
Δρ_max_ = 0.62 e Å^−3^
Δρ_min_ = −0.30 e Å^−3^

### 

Data collection: *APEX2* (Bruker, 2005 ▶); cell refinement: *SAINT* (Bruker, 2001 ▶); data reduction: *SAINT*; program(s) used to solve structure: *SHELXTL* (Sheldrick, 2008 ▶); program(s) used to refine structure: *SHELXTL*; molecular graphics: *SHELXTL*; software used to prepare material for publication: *SHELXTL*.

## Supplementary Material

Crystal structure: contains datablock(s) global, I. DOI: 10.1107/S1600536813019545/cv5422sup1.cif

Structure factors: contains datablock(s) I. DOI: 10.1107/S1600536813019545/cv5422Isup2.hkl

Supplementary material file. DOI: 10.1107/S1600536813019545/cv5422Isup3.cml

Additional supplementary materials:  crystallographic information; 3D view; checkCIF report

## Figures and Tables

**Table 1:** Hydrogen-bond geometry (Å, °) *Cg* is the centroid of C6–C11 ring.

*D*—H⋯*A*	*D*—H	H⋯*A*	*D*⋯*A*	*D*—H⋯*A*
C3—H3⋯*Cg* ^i^	0.95	2.60	3.483 (2)	158

Symmetry code: (i) 
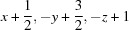
.

**Fig. 1.**
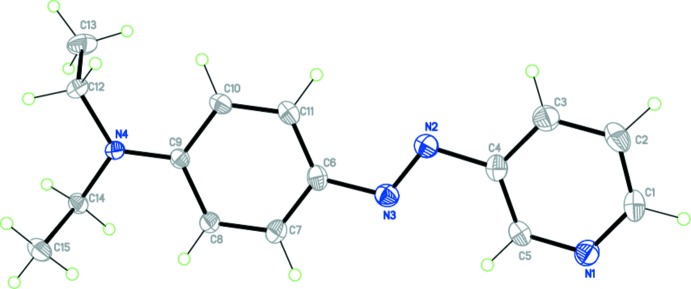
Molecular structure of I. Displacement ellipsoids are shown at the 50% probability level. H atoms are presented as small spheres of arbitrary radius.

**Fig. 2.**
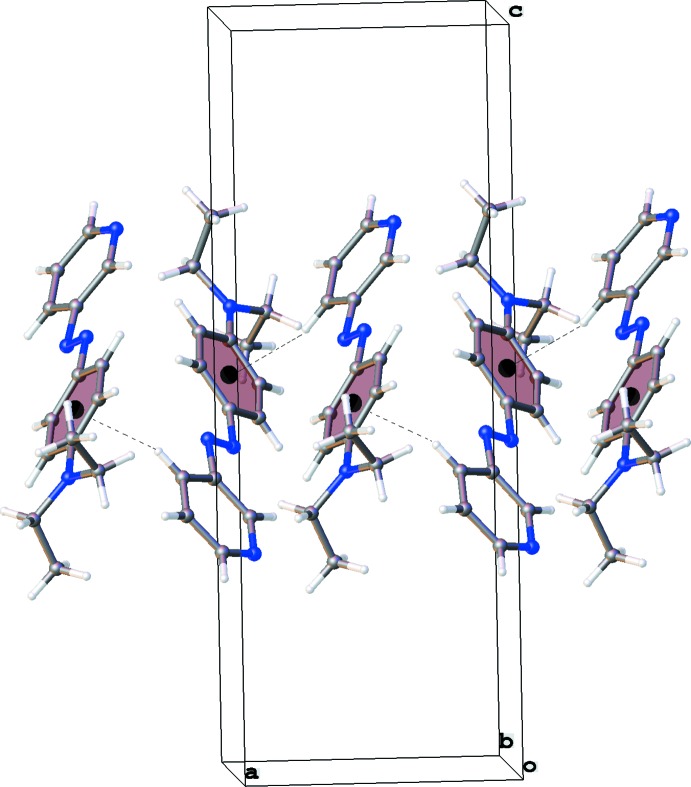
A portion of the crystal packing, showing the weak C—H···π interactions by dashed lines.

## supplementary crystallographic information

### Comment

This molecule as a donor-acceptor substituted stilbene-like derivative supposed to show nonlinear optical response (Forrest *et al.*, 1996). According to experimental data, such a response for molecules with a CH═CH bridge is higher than for molecules with an N═N bridge (Chemla & Zyss, 1987). On the other hand, theoretical calculations have predicted that azobenzenes can exhibit larger hyperpolarizabilities than stilbene analogues (Morley, 1995). The title compound has a noncentrosymmetric packing and therefore crystals of this material might demonstrate SHG. Usually because of antiparallel dipole positions in the *P*2_1_2_1_2_1_ space group the SHG is weak or undetectable. However there are some exceptions from such regularity, for instance, for molecules of coordination compounds described with two dipole moments (Rivera *et al.*, 2006) SHG was experimentally observed. We tried to experimentally evaluate SHG of this crystal. SHG experiment on a single crystal was done; laser power was increased step by step without appearance of visible SHG. At the maximal laser power crystals were melted under femtosecond laser beam. The absence of SHG for tested sample is not surprising since orientation of neighboring molecules in crystal is antiparallel that prevents SHG.

The molecular structure of the title compound (I) (Fig. 1) shows the presence of an N═N [1.2856 (2) A] double bond; the molecule adopts almost planar *Z*-configuration with the dihedral angle between the two aromatic rings equal to 8.03 (5)°. The endocyclic angles of pyridine ring cover the range 116.64 (5)–124.09 (5)°. The endocyclic angles at the C1 and C5 atoms adjacent to the N1 heteroatom are larger than 120°, and those at the other atoms of the ring are smaller than 120°. Same distribution of endocyclic angles was observed in the other pyridine compounds reported by us earlier (Draguta *et al.*, 2012). The C9—N4, C6—N3 and C4—N2 bond lengths are 1.3666 (15), 1.4213 (17) and 1.4284 (17) Å, respectively, consistent with the single and double bonds between related C and N atoms.

In the absence of hydrogen bonds and stacking, crystal packing of title compound is determined by weak C—H···π (Table 1, Fig. 2) interactions which stabilize herringbone motif into antiparallel molecular orientation, with the angle between molecular vectors connecting C1 and N4 atoms equal to 178.00 (2)°.

### Experimental

Title compound was synthesized according to the published procedure (Peor *et al.*, 2008). After purification red plate-like crystals with melting point of 114°C were obtained from slow evaporation from ethanol solution. Second harmonic generation (SHG) in single-crystal of the compound under investigation was tested using irradiation by laser beam with diameter 2 mm, wavelength 1.04 µm, power 700 mW, duration 150 fs at 75 MHz repetition. Initial power density was 2 × 10 ^6^ W cm^-2^; it was increased step by step up to melting of the sample. UV–Vis: 385 nm; fluorescence: 480 nm.

### Refinement

H atoms attached to C atoms were found in difference Fourier maps and subsequently placed at calculated positions with C—H = 0.95 Å (aromatic) or 0.98–0.99 Å (sp^3^ C atom). Isotropic displacement parameters for these H atoms were calculated as *U*iso(H) = 1.5*U*eq(carrier C) in the case of the methyl group, and *U*iso(H) = 1.2*U*eq(carrier C) otherwise. Since this is a light-atom structure determined with Mo Kα radiation, there is no anomalous signal with which to refine a meaningful Flack parameter. For this reason, 895 Friedel pairs were merged for the final rounds of refinement.

### Crystal data

**Table:**

C_15_H_18_N_4_	*F*(000) = 544
*M**_r_* = 254.33	*D*_x_ = 1.235 Mg m^−^^3^
Orthorhombic, *P*2_1_2_1_2_1_	Mo *K*α radiation, λ = 0.71073 Å
Hall symbol: P 2ac 2ab	Cell parameters from 8453 reflections
*a* = 7.4332 (7) Å	θ = 4.5–30.6°
*b* = 9.1093 (8) Å	µ = 0.08 mm^−^^1^
*c* = 20.1946 (19) Å	*T* = 100 K
*V* = 1367.4 (2) Å^3^	Plate, red
*Z* = 4	0.30 × 0.25 × 0.20 mm

### Data collection

**Table:**

Bruker APEXII CCD diffractometer	4195 independent reflections
Radiation source: fine-focus sealed tube	4012 reflections with *I* > 2σ(*I*)
Graphite monochromator	*R*_int_ = 0.022
φ and ω scans	θ_max_ = 30.7°, θ_min_ = 4.5°
Absorption correction: multi-scan (*SADABS*; Sheldrick, 2003)	*h* = −10→10
*T*_min_ = 0.977, *T*_max_ = 0.985	*k* = −13→13
16318 measured reflections	*l* = −28→28

### Refinement

**Table:**

Refinement on *F*^2^	Primary atom site location: structure-invariant direct methods
Least-squares matrix: full	Secondary atom site location: difference Fourier map
*R*[*F*^2^ > 2σ(*F*^2^)] = 0.046	Hydrogen site location: inferred from neighbouring sites
*wR*(*F*^2^) = 0.113	H-atom parameters constrained
*S* = 1.00	*w* = 1/[σ^2^(*F*_o_^2^) + (0.050*P*)^2^ + 0.7002*P*] where *P* = (*F*_o_^2^ + 2*F*_c_^2^)/3
4195 reflections	(Δ/σ)_max_ = 0.001
174 parameters	Δρ_max_ = 0.62 e Å^−^^3^
0 restraints	Δρ_min_ = −0.30 e Å^−^^3^

### Special details

**Table:**

Geometry. All e.s.d.'s (except the e.s.d. in the dihedral angle between two l.s. planes) are estimated using the full covariance matrix. The cell e.s.d.'s are taken into account individually in the estimation of e.s.d.'s in distances, angles and torsion angles; correlations between e.s.d.'s in cell parameters are only used when they are defined by crystal symmetry. An approximate (isotropic) treatment of cell e.s.d.'s is used for estimating e.s.d.'s involving l.s. planes.
Refinement. Refinement of *F*^2^ against ALL reflections. The weighted *R*-factor *wR* and goodness of fit *S* are based on *F*^2^, conventional *R*-factors *R* are based on *F*, with *F* set to zero for negative *F*^2^. The threshold expression of *F*^2^ > σ(*F*^2^) is used only for calculating *R*-factors(gt) *etc*. and is not relevant to the choice of reflections for refinement. *R*-factors based on *F*^2^ are statistically about twice as large as those based on *F*, and *R*-factors based on ALL data will be even larger.

### Fractional atomic coordinates and isotropic or equivalent isotropic displacement parameters (Å^2^)

**Table:**

	*x*	*y*	*z*	*U*_iso_*/*U*_eq_	
N4	0.00601 (15)	0.19116 (12)	0.62882 (5)	0.0171 (2)	
C9	0.00202 (16)	0.29808 (13)	0.58137 (6)	0.0156 (2)	
C4	0.02116 (18)	0.83461 (14)	0.38104 (7)	0.0203 (2)	
C8	−0.10014 (18)	0.27948 (14)	0.52266 (6)	0.0188 (2)	
H8	−0.1662	0.1914	0.5161	0.023*	
C10	0.09862 (17)	0.43213 (14)	0.58851 (6)	0.0190 (2)	
H10	0.1695	0.4479	0.6270	0.023*	
N3	−0.03161 (16)	0.61944 (13)	0.42929 (6)	0.0214 (2)	
C7	−0.10486 (19)	0.38750 (15)	0.47499 (7)	0.0214 (2)	
H7	−0.1736	0.3720	0.4360	0.026*	
N2	0.04908 (15)	0.73981 (13)	0.43644 (6)	0.0216 (2)	
N1	−0.12281 (17)	0.90062 (14)	0.27805 (6)	0.0252 (2)	
C6	−0.01126 (18)	0.51897 (14)	0.48261 (7)	0.0202 (2)	
C15	−0.03051 (19)	−0.06308 (14)	0.58509 (7)	0.0232 (3)	
H15A	−0.0141	−0.0317	0.5391	0.035*	
H15B	−0.1116	−0.1479	0.5865	0.035*	
H15C	0.0862	−0.0904	0.6040	0.035*	
C14	−0.11139 (17)	0.06233 (14)	0.62505 (6)	0.0182 (2)	
H14A	−0.2272	0.0915	0.6047	0.022*	
H14B	−0.1369	0.0272	0.6705	0.022*	
C3	0.1248 (2)	0.96131 (17)	0.37904 (8)	0.0271 (3)	
H3	0.2076	0.9829	0.4135	0.032*	
C11	0.09094 (18)	0.54000 (14)	0.54022 (7)	0.0202 (2)	
H11	0.1555	0.6290	0.5462	0.024*	
C1	−0.0180 (2)	1.02026 (16)	0.27692 (7)	0.0263 (3)	
H1	−0.0288	1.0849	0.2402	0.032*	
C5	−0.10281 (19)	0.81021 (15)	0.32994 (7)	0.0217 (2)	
H5	−0.1766	0.7252	0.3322	0.026*	
C12	0.13549 (18)	0.19273 (15)	0.68329 (6)	0.0201 (2)	
H12A	0.2440	0.2474	0.6692	0.024*	
H12B	0.1724	0.0906	0.6931	0.024*	
C2	0.1047 (2)	1.05542 (16)	0.32577 (8)	0.0292 (3)	
H2	0.1741	1.1427	0.3229	0.035*	
C13	0.0618 (2)	0.2623 (2)	0.74616 (7)	0.0303 (3)	
H13A	0.0375	0.3666	0.7382	0.045*	
H13B	0.1504	0.2525	0.7818	0.045*	
H13C	−0.0500	0.2128	0.7590	0.045*	

### Atomic displacement parameters (Å^2^)

**Table:**

	*U*^11^	*U*^22^	*U*^33^	*U*^12^	*U*^13^	*U*^23^
N4	0.0193 (5)	0.0161 (4)	0.0160 (4)	−0.0027 (4)	−0.0034 (4)	0.0008 (4)
C9	0.0150 (5)	0.0145 (5)	0.0173 (5)	0.0002 (4)	0.0011 (4)	−0.0009 (4)
C4	0.0194 (6)	0.0191 (5)	0.0224 (6)	0.0049 (5)	0.0038 (5)	0.0017 (4)
C8	0.0185 (5)	0.0175 (5)	0.0205 (5)	−0.0005 (4)	−0.0030 (5)	0.0011 (4)
C10	0.0178 (5)	0.0164 (5)	0.0228 (5)	−0.0006 (5)	−0.0006 (5)	−0.0031 (4)
N3	0.0187 (5)	0.0212 (5)	0.0241 (5)	−0.0001 (4)	−0.0010 (4)	−0.0014 (4)
C7	0.0207 (6)	0.0224 (6)	0.0211 (5)	0.0028 (5)	−0.0025 (5)	0.0023 (5)
N2	0.0196 (5)	0.0210 (5)	0.0241 (5)	0.0008 (4)	−0.0007 (4)	−0.0009 (4)
N1	0.0250 (6)	0.0263 (6)	0.0241 (5)	−0.0002 (5)	−0.0011 (5)	0.0021 (5)
C6	0.0187 (6)	0.0176 (5)	0.0242 (6)	0.0022 (5)	0.0026 (5)	0.0028 (4)
C15	0.0217 (6)	0.0174 (5)	0.0305 (6)	−0.0012 (5)	−0.0006 (5)	−0.0037 (5)
C14	0.0181 (5)	0.0157 (5)	0.0208 (5)	−0.0028 (4)	−0.0003 (4)	0.0016 (4)
C3	0.0237 (6)	0.0271 (7)	0.0305 (7)	−0.0012 (6)	−0.0042 (6)	0.0011 (6)
C11	0.0188 (6)	0.0156 (5)	0.0262 (6)	−0.0004 (5)	0.0021 (5)	−0.0004 (4)
C1	0.0253 (6)	0.0242 (6)	0.0295 (7)	0.0016 (5)	0.0042 (6)	0.0097 (5)
C5	0.0217 (6)	0.0169 (5)	0.0266 (6)	−0.0001 (5)	0.0020 (5)	0.0004 (5)
C12	0.0209 (6)	0.0220 (6)	0.0174 (5)	−0.0008 (5)	−0.0050 (4)	0.0003 (4)
C2	0.0240 (6)	0.0221 (6)	0.0414 (8)	−0.0046 (5)	0.0011 (6)	0.0038 (6)
C13	0.0338 (8)	0.0389 (8)	0.0183 (6)	−0.0062 (7)	−0.0009 (5)	−0.0064 (6)

### Geometric parameters (Å, º)

**Table:**

N4—C9	1.3665 (15)	C15—C14	1.5224 (18)
N4—C14	1.4645 (16)	C15—H15A	0.9800
N4—C12	1.4616 (16)	C15—H15B	0.9800
C9—C8	1.4182 (17)	C15—H15C	0.9800
C9—C10	1.4239 (17)	C14—H14A	0.9900
C4—C3	1.388 (2)	C14—H14B	0.9900
C4—C5	1.4012 (19)	C3—C2	1.384 (2)
C4—N2	1.4285 (17)	C3—H3	0.9500
C8—C7	1.3770 (18)	C11—H11	0.9500
C8—H8	0.9500	C1—C2	1.381 (2)
C10—C11	1.3856 (18)	C1—H1	0.9500
C10—H10	0.9500	C5—H5	0.9500
N3—N2	1.2581 (16)	C12—C13	1.5212 (19)
N3—C6	1.4213 (17)	C12—H12A	0.9900
C7—C6	1.3936 (19)	C12—H12B	0.9900
C7—H7	0.9500	C2—H2	0.9500
N1—C1	1.3398 (19)	C13—H13A	0.9800
N1—C5	1.3411 (18)	C13—H13B	0.9800
C6—C11	1.4027 (19)	C13—H13C	0.9800
			
C9—N4—C14	121.45 (10)	C15—C14—H14A	108.9
C9—N4—C12	122.34 (11)	N4—C14—H14B	108.9
C14—N4—C12	116.06 (10)	C15—C14—H14B	108.9
N4—C9—C8	120.84 (11)	H14A—C14—H14B	107.8
N4—C9—C10	121.95 (11)	C4—C3—C2	118.55 (14)
C8—C9—C10	117.20 (11)	C4—C3—H3	120.7
C3—C4—C5	118.38 (12)	C2—C3—H3	120.7
C3—C4—N2	116.42 (12)	C10—C11—C6	120.61 (12)
C5—C4—N2	125.20 (12)	C10—C11—H11	119.7
C7—C8—C9	120.85 (12)	C6—C11—H11	119.7
C7—C8—H8	119.6	N1—C1—C2	124.08 (13)
C9—C8—H8	119.6	N1—C1—H1	118.0
C11—C10—C9	121.08 (12)	C2—C1—H1	118.0
C11—C10—H10	119.5	N1—C5—C4	123.43 (13)
C9—C10—H10	119.5	N1—C5—H5	118.3
N2—N3—C6	115.05 (11)	C4—C5—H5	118.3
C8—C7—C6	121.61 (12)	N4—C12—C13	113.27 (12)
C8—C7—H7	119.2	N4—C12—H12A	108.9
C6—C7—H7	119.2	C13—C12—H12A	108.9
N3—N2—C4	111.57 (11)	N4—C12—H12B	108.9
C1—N1—C5	116.64 (13)	C13—C12—H12B	108.9
C7—C6—C11	118.64 (12)	H12A—C12—H12B	107.7
C7—C6—N3	114.62 (12)	C1—C2—C3	118.88 (14)
C11—C6—N3	126.74 (12)	C1—C2—H2	120.6
C14—C15—H15A	109.5	C3—C2—H2	120.6
C14—C15—H15B	109.5	C12—C13—H13A	109.5
H15A—C15—H15B	109.5	C12—C13—H13B	109.5
C14—C15—H15C	109.5	H13A—C13—H13B	109.5
H15A—C15—H15C	109.5	C12—C13—H13C	109.5
H15B—C15—H15C	109.5	H13A—C13—H13C	109.5
N4—C14—C15	113.18 (11)	H13B—C13—H13C	109.5
N4—C14—H14A	108.9		
			
C14—N4—C9—C8	−7.75 (18)	C9—N4—C14—C15	87.55 (14)
C12—N4—C9—C8	167.67 (12)	C12—N4—C14—C15	−88.15 (14)
C14—N4—C9—C10	172.05 (11)	C5—C4—C3—C2	−1.9 (2)
C12—N4—C9—C10	−12.53 (18)	N2—C4—C3—C2	178.95 (13)
N4—C9—C8—C7	179.69 (12)	C9—C10—C11—C6	−0.63 (19)
C10—C9—C8—C7	−0.12 (18)	C7—C6—C11—C10	0.04 (19)
N4—C9—C10—C11	−179.14 (12)	N3—C6—C11—C10	179.91 (12)
C8—C9—C10—C11	0.66 (18)	C5—N1—C1—C2	−1.0 (2)
C9—C8—C7—C6	−0.5 (2)	C1—N1—C5—C4	−0.7 (2)
C6—N3—N2—C4	−179.69 (11)	C3—C4—C5—N1	2.1 (2)
C3—C4—N2—N3	−170.84 (12)	N2—C4—C5—N1	−178.75 (13)
C5—C4—N2—N3	10.04 (18)	C9—N4—C12—C13	95.10 (15)
C8—C7—C6—C11	0.5 (2)	C14—N4—C12—C13	−89.25 (14)
C8—C7—C6—N3	−179.38 (12)	N1—C1—C2—C3	1.2 (2)
N2—N3—C6—C7	178.43 (12)	C4—C3—C2—C1	0.3 (2)
N2—N3—C6—C11	−1.45 (19)		

### Hydrogen-bond geometry (Å, º)

Cg is the centroid of C6–C11 ring.

**Table:**

*D*—H···*A*	*D*—H	H···*A*	*D*···*A*	*D*—H···*A*
C3—H3···*Cg*^i^	0.95	2.60	3.483 (2)	158

Symmetry code: (i) *x*+1/2, −*y*+3/2, −*z*+1.
